# Modulation of intracortical circuits in primary motor cortex during automatic action tendencies

**DOI:** 10.1007/s00429-024-02783-7

**Published:** 2024-03-14

**Authors:** Xue Xia, Yansong Li, Yuyu Song, Yuanjun Dong, Robert Chen, Jian Zhang, Xiaoying Tan

**Affiliations:** 1 School of Social Development and Health Management, University of Health and Rehabilitation Sciences, Qingdao, China; 2 School of Psychology, Shanghai University of Sport, Shanghai, China; 3 School of Physical Education, Qingdao University, Qingdao, China; 4 Krembil Research Institute, University Health Network, Toronto, Canada; 5 Division of Neurology, Department of Medicine, University of Toronto, Toronto, Canada; 6 Faculty of Health Sciences and Sports, Macao Polytechnic University, Rua de Luis Gonzaga Gomes, Macao S.A.R., China

**Keywords:** Primary motor cortex, Short-interval intracortical inhibition, Intracortical facilitation, Automatic action tendency, Manikin task, Paired-pulse transcranial magnetic stimulation

## Abstract

Humans display automatic action tendencies toward emotional stimuli, showing faster automatic behavior (i.e., approaching a positive stimulus and avoiding a negative stimulus) than regulated behavior (i.e., avoiding a positive stimulus and approaching a negative stimulus). Previous studies have shown that the primary motor cortex is involved in the processing of automatic actions, with higher motor evoked potential amplitudes during automatic behavior elicited by single-pulse transcranial magnetic stimulation. However, it is unknown how intracortical circuits are involved with automatic action tendencies. Here, we measured short-interval intracortical inhibition and intracortical facilitation within the primary motor cortex by using paired-pulse transcranial magnetic stimulation protocols during a manikin task, which has been widely used to explore approaching and avoiding behavior. Results showed that intracortical facilitation was stronger during automatic behavior than during regulated behavior. Moreover, there was a significant negative correlation between reaction times and intracortical facilitation effect during automatic behavior: individuals with short reaction times had stronger faciliatory activity, as shown by higher intracortical facilitation. By contrast, no significant difference was found for short-interval intracortical inhibition between automatic behavior and regulated behavior. The results indicated that the intracortical facilitation circuit, mediated by excitatory glutamatergic neurons, in the primary motor cortex, plays an important role in mediating automatic action tendencies. This finding further supports the link between emotional perception and the action system.

## Introduction

Emotional stimuli have a substantial impact on our daily lives, and the ability to rapidly recognize and react to emotional stimuli is crucial for survival and social interactions. As a typical cognitive bias, emotion-induced automatic action tendency triggers a quick approach toward positive stimuli and an avoidance of negative stimuli when a rapid response to emotional stimuli is required in the short term (Chen and Bargh 1999). On the other hand, regulation of the immediate approaching-avoiding tendencies with behaviors in the opposite direction is often observed for achieving long-term goals in humans, with a regulated approach toward negative stimuli and an avoidance of positive stimuli (Ernst et al. 2013a, b). However, such regulated behaviors may not be carried out simply because they depend on both the successful suppression of the automatic response and the launch of an opposite behavior (Berkman and Lieberman 2010). Failure to execute the regulated behavior may result in abnormal motivational behavior, such as automatic approaching toward drugs, alcohol, or high-calorie foods by individuals with drug or alcohol use disorders or eating disorders, respectively. Thus, it is important to investigate how automatic and regulated approaching-avoiding behaviors occur, to provide a theoretical basis for the cause of abnormal approaching-avoiding behavior.

The primary motor cortex (M1), which plays a crucial role in the execution of voluntary movements, has also been shown to play a key role in higher cognitive processes (Bhattacharjee et al. 2021). Studies assessing automatic action tendencies have reported that M1 excitability is greater during automatic behavior (Fini et al. 2020; Fischer et al. 2020; Xia et al. 2021b) but that dorsolateral prefrontal cortex–M1 connectivity is stronger during regulated behavior (Xia et al. 2022). Those studies have described a portion of the underlying neurophysiological mechanisms; however, how intracortical neural circuits within M1 respond remains unclear. What is known is that synaptic excitation and inhibition are inextricably linked: the relative strength and timing of each excitatory and inhibitory event contribute to the neuronal outputs that ultimately regulate cortical function (Isaacson and Scanziani 2011; Tatti et al. 2017). Improvement in response performance is also mediated by intracortical circuits, such as a faster response accompanied by the release of intracortical inhibition and the enhancement of intracortical excitation (Davranche et al. 2007; Tandonnet et al. 2010). The purpose of the present study was to explore how the inhibitory and excitatory circuits of neurons in the cortex of M1 participate in automatic action tendencies to further reveal the processing mechanisms of this effect and to better understand cortical function.

Transcranial magnetic stimulation (TMS) has the advantage of high temporal resolution and could be an effective tool to measure the M1 activities, indexed by motor evoked potentials (MEPs) induced in a target muscle (Hallett 2000). Intracortical excitation and inhibition can be measured by using a paired-pulse TMS protocol with a subthreshold conditioning stimulus (CS) followed by a suprathreshold test stimulus (TS) at specific interstimulus intervals applied to M1 through the same coil (Kujirai et al. 1993). Short-interval intracortical inhibition (SICI), which primarily reflects the gamma-aminobutyric acid (GABA)-ergic inhibition, and intracortical facilitation (ICF), which is thought to be affected by glutamatergic facilitation through N-methyl-D-aspartate (NMDA) receptors, are widely used markers of M1 cortical circuits (Ziemann et al. 1996). They are modulated by cognitive processes, such as emotional perception and response inhibition (Borgomaneri et al. 2015a; Chowdhury et al. 2019a). Viewing emotional body expressions, especially fearful facial expressions, suppresses ICF compared with the viewing of neutral expressions, suggesting that emotional signals lead to defensive suppression of M1 excitatory activity (Borgomaneri et al. 2015b, 2017). SICI is related to the ability to restrain and cancel behavior, with individuals who are faster at stopping actions showing a greater degree of SICI in M1 (Chowdhury et al. 2018, 2019a, b; He et al. 2019).

The purpose of the current study was to investigate changes in intracortical circuits associated with automatic action tendencies by examining SICI and ICF during a manikin task, which has been widely used for assessing automatic approaching and avoiding tendencies (Krieglmeyer and Deutsch 2010). For the manikin task used in the present study, participants moved an image of their own face that was displayed on a computer screen through the use of instructed key presses toward or away from an emotional image that was also shown on the screen. Participants could display four types of behavior, namely, approaching or avoiding behavior toward positive or negative stimuli (Xia et al. 2021a, b, 2022, 2023). Given that previous studies have found lower ICF during the perception of either happy or fearful signals compared with neutral signals (Borgomaneri et al. 2015b, 2017), we hypothesized that stronger ICF would occur during an automatic behavior to facilitate a behavior that is consistent with the automatic action tendency. Because SICI has been shown to be involved in response inhibition, we hypothesized that stronger SICI would occur during a regulated behavior to inhibit the automatic behavior that was induced by the automatic action tendency.

## Methods

The present study used approaches similar to those in our prior publications (Xia et al. 2021b, 2022, 2023). Consequently, some text included here and in other sections is recycled from those sources.

### Participants

Power analyses based on a medium effect (effect size *f* = 0.25) yielded a sample size of 20 with an error probability of 0.05 and a power of 90%, as calculated using G*Power 3.1 (Faul et al. 2007). A total of 20 right-handed students [10 females; mean (SD) age, 20.7 (1.9) years; age range, 18–24 years] were recruited from Shanghai University of Sport. Inclusion criteria were normal or corrected-to-normal vision and no history of psychiatric or neurological disorders or contraindication for undergoing TMS (Keel et al. 2001). Participants provided written informed consent prior to beginning the study. The Ethics Committee of Shanghai University of Sport approved this study protocol, and the Declaration of Helsinki tenets were followed.

### TMS protocol

A figure-eight coil (70 mm, D70 Alpha Flat Coi, Magstim) connected to a Magstim Bistim^2^ module (Magstim, Whitland, Dyfed, U.K.), which could generate two output pulses by two Magstim 200 stimulators, was used. The coil was placed over the left M1, with the handle of the coil pointed backward at an angle 30°–45° to the midsagittal line and induced a posterior-anterior directed current. The M1 stimulation hotspot was defined as the location where a given suprathreshold stimulation produced the largest MEP amplitude in the target muscle. SICI and ICF were measured using a paired-pulse TMS protocol, with a CS followed by a TS applied to M1 through the same coil (Kujirai et al. 1993). We recorded MEPs as participants performed the manikin task under three TMS conditions: TS alone, SICI, and ICF. The TS intensity was that which evoked approximately 1.0-mV MEPs recorded from the first dorsal interosseous (FDI) muscle. The CS intensity used for the SICI and ICF conditions was 80% of the resting motor threshold. The resting motor threshold was determined by assessing the minimum intensity in at least 5 of 10 successive trials that would induce MEPs with an amplitude >50 μV in the FDI muscle while it was relaxed. To assess SICI circuits, short interstimulus intervals of 3 ms were used, whereas 12 ms were used to assess ICF circuits (Borgomaneri et al. 2015b). The TS occurred 200 ms after stimulus onset, when higher MEP amplitudes have been observed for automatic behavior compared with regulated behavior (Xia et al. 2021b).

### Electromyography (EMG)

Active, reference, and ground disposable surface electrodes were fixed over the right FDI muscle belly, the metacarpophalangeal joint, and the wrist, respectively. The EMG signal was × 1000 amplified using an Intronix amplifier (Model 2024F, Intronix Technologies, Ontario, Canada), 20 Hz-2.5 kHz bandpass filtered, and digitally sampled at a frequency of 5 kHz with Micro 1201-4 data acquisition unit (Cambridge Electronics Design, Cambridge, UK). Data were later analyzed offline.

### Manikin task

**Fig. 1:**
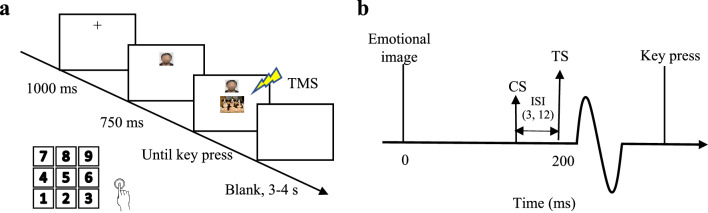
Experimental setup. **a** Example of a trial sequence in the manikin task. **b** The stimulus configurations of TMS

We used MATLAB software to conduct the manikin task as previously described (Xia et al. 2021b). The procedure was shown in the Fig. 1. Briefly, a fixation cross was displayed for 1000 ms either in the top or bottom half of the computer screen with a 50% probability for each location, followed by an image of the participant’s own face displayed for 750 ms at that same location. Either a negative or positive emotional image was then displayed in the center of the screen. To move their face image to approach or avoid the positive or negative image, participants pressed “8” on a numeric keypad with the right index finger to move their face image up or pressed “2” to move it down. Once the participant had responded, the screen was blank for 3–4 s. This additional time was introduced to ensure that the TMS interpulse interval was >5 s to avoid potential confounding of changes in motor excitability caused by TMS itself. In the automatic behavior block, participants were asked to approach a positive image and avoid a negative image, whereas the instructions were reversed for the regulated behavior block. The order of these two blocks was counterbalanced across participants. For both automatic and regulated blocks, 72 trials in random order were collected, with 12 trials for each TMS condition (TS alone, SICI and ICF) and for each behavior (automatic block: approaching positive images and avoiding negative behavior; regulated block: avoiding positive images and approaching negative images). Twelve trials for practice were performed before each block. Before the manikin task began, we recorded a baseline of 12 MEPs at the TS intensity while participants were at rest.

### Emotional images

The 36 images selected for the present study from the International Affective Image System (Lang et al. 1999) were the same as those used in our previous publication (Xia et al. 2022). Among them, 18 images were considered positive (*M*_valence_ = 7.72, *M*_arousal_ = 5.31), for example, happy, smiling faces and flowers, and 18 images were negative (*M*_valence_ = 2.58, *M*_arousal_ = 5.47), for example, images considered disgusting or threatening, such as a snake. Paired t-tests of the two groups of images showed a significant difference in valence [*t* (17) = 36.67, *p* < 0.001], but not in arousal [*t* (17) = 0.98, *p* = 0.34]. Each image appeared twice in each block.

### Data and statistical analyses

Reaction times (RTs) were recorded by MATLAB. The RT was defined as the time between the display of the emotional image and the key press. For MEP data, we extracted peak-to-peak amplitudes in SIGNAL 6.0 software (Cambridge Electronics Design, Cambridge, UK). Trials meeting the following criteria were included for further analysis: (1) the response was correct, (2) the RT was within three times the standard deviation (SD) of the mean RT, and (3) 100 ms before the onset of TMS pulse, the root mean square of background EMG activity was within ±2 SDs of the mean root mean square EMG (Xia et al. 2021b).

We determined corticospinal excitability during approaching or avoiding behavior by calculating the ratio of the mean MEP amplitude during the task to baseline (% of baseline) for the three TMS conditions. We compared corticospinal excitability and RTs during each behavior for the three TMS conditions using a three-way repeated measures (RM) analysis of variance (ANOVA) for RTs and MEP ratios (% of baseline). Emotion (positive, negative), behavior (approaching, avoiding), and TMS condition (TS alone, SICI, and ICF) were considered within-subject factors. It should be noted that the RM ANOVA of MEP ratios (% of baseline) can only confirm the direction of the paired-pulse protocol, with expected lower MEPs when the CS preceded the TS by 3 ms and larger MEPs when the CS preceded the TS by 12 ms. However, this analysis did not enable us to rule out a possible contribution of spinal excitability because it was standardized by the baseline MEP that was collected at rest (Borgomaneri et al. 2015b). To quantify ICF and SICI effects, we expressed MEPs in the SICI/ICF condition relative to the TS alone condition (% of TS alone) to estimate the effects of the subthreshold CS on the MEP elicited by the suprathreshold TS. We used a three-way RM ANOVA for MEP ratios (% of TS alone) using emotion, behavior, and TMS condition (SICI vs. ICF) as within-subject factors. When interactions in the RM ANOVA were statistically significant, paired-sample t-tests with Bonferroni corrections were performed for post-hoc analyses to examine differences between approaching and avoiding behaviors at each TMS condition (Borgomaneri et al. 2015b, 2017).

To directly compare the automatic and regulated block, the RT and MEP ratio of the two types of behavior in each block were averaged. We performed two-way RM ANOVA for the averaged RT and MEP ratio (% of baseline), using block (automatic vs. regulated) and TMS condition (TS alone, SICI, and ICF) as within-subject factors. Another two-way RM ANOVA was conducted for MEP ratio (% of TS alone), with block (automatic vs. regulated) and TMS condition (SICI vs. ICF) as within-subject factors.

Finally, Pearson correlations between RTs and MEP ratios were analyzed for the TMS conditions that showed significantly different modulations for the automatic and regulated behaviors. Violations of sphericity were handled using the Greenhouse–Geisser correction (Mauchly’s test statistic <0.05). Values of *p* < 0.05 were considered statistically significant. Data are shown as mean ± SD.

## Results

### Reaction times

**Fig. 2:**
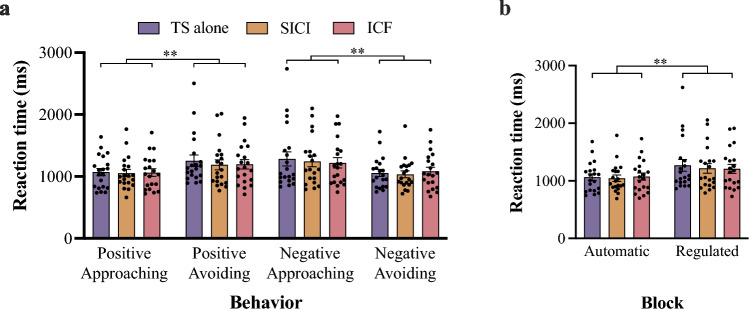
Reaction times for each TMS condition sorted by behavior (**a**) and block (**b**). *Error bars* indicate standard errors of means (SEMs). ^**^p < 0.01

A three-way RM ANOVA assessing RTs indicated that the main effects were not significant [emotion:* F*(1, 19) = 1.20, *p* = 0.287, ${\eta }_{p}^{2}$ = 0.06; behavior: *F*(1, 19) = 1.88, *p* = 0.186, ${\eta }_{p}^{2}$ = 0.09; TMS condition: *F*(1.349, 25.640) = 0.79, *p* = 0.418, ${\eta }_{p}^{2}$ = 0.04]. By contrast, there was a significant interaction between emotion and behavior [*F*(1, 19) = 12.07, *p* = 0.003, ${\eta }_{p}^{2}$ = 0.39]. Post-hoc tests indicated shorter RTs for approaching positive image (1062.3 ± 225.7 ms) than for avoiding [1212.6 ± 360.7 ms, *p* = 0.003, *d* = 0.48, 95% CI = (58.7, 241.9)], and shorter RTs for avoiding negative image (1058.4 ± 250.7 ms) than for approaching [1248.8 ± 406.4 ms, *p* = 0.004,* d* = 0.56, 95% CI = (69.8, 311.1)]. The RTs of each behavior are shown in Fig. 2a.

The RTs sorted by block are shown in Fig. 2b. The results of a two-way RM ANOVA showed a significant main effect of block [*F*(1, 19) = 12.07, *p* = 0.003, ${\eta }_{p}^{2}$ = 0.39], with the post-hoc analysis indicating that the RT of the automatic block (1060.4 ± 250.3 ms) was significantly shorter than that of the regulated block [1230.7 ± 380.5 ms; *d* = 0.53, 95% CI = (67.7, 272.0)]. No other significant main effects or interactions were detected.

### Corticospinal excitability

**Fig. 3:**
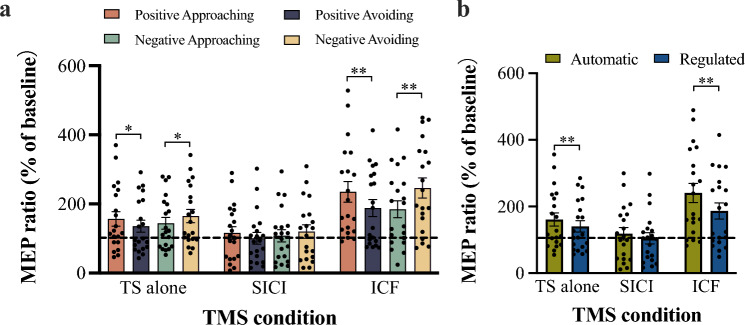
MEP ratio (% of baseline) for each TMS condition sorted by behavior (**a**) and block (**b**). *Error bars* indicate SEM. The *dashed line* indicates the MEP ratio at baseline (100%). Ratios <100% indicate inhibition, and ratios >100% indicate facilitation. ^*^p < 0.05; ^**^p < 0.01

The mean TMS intensity needed to generate an approximately 1.0-mV MEP was 49.8% of the maximum stimulator output (range, 38–74%). The MEP amplitude recorded at rest which served as baseline was 1.02 ± 0.34 mV. The MEP amplitudes for three TMS conditions are shown in Table 1 and the MEP ratios (% of baseline) are shown in Fig. 3a. The results of a three-way ANOVA of MEP ratios showed a significant main effect of TMS condition [*F*(1.424, 27.047) = 34.94, *p* < 0.001, ${\eta }_{p}^{2}$ = 0.65]. Post-hoc analyses indicated that the MEP ratio under the ICF condition (213.6 ± 113.8%) was higher than those for the TS alone condition [150.5 ± 80.8%; *p* < 0.001, *d* = 0.64, 95% CI = (33.2, 93.0)] and the SICI condition [111.3 ± 77.7%; *p* < 0.001, *d* = 1.05, 95% CI = (61.2, 143.5)]; and that the MEP ratio under the SICI condition was lower than that for the TS alone condition [*p* = 0.001, *d* = 0.50, 95% CI = (15.4, 63.0)]. The main effects of emotion and behavior were not significant. The two-way interaction between emotion and behavior was significant [*F*(1, 19) = 16.4, *p* = 0.001, ${\eta }_{p}^{2}$ = 0.46], and the three-way interaction was also significant [*F*(1.342, 25.499) = 5.67, *p* = 0.017, ${\eta }_{p}^{2}$ = 0.23]. Post-hoc analyses indicated that under the TS alone condition and the ICF condition, the MEP ratio for the positive approaching behavior was significantly higher than that for the positive avoiding behavior [TS alone condition: *p* = 0.040, *d* = 0.24, 95% CI = (1.1, 41.1); ICF condition: *p* = 0.005, *d* = 0.39, 95% CI = (16.1, 77.6)]; and the MEP ratio for negative avoiding behavior was significantly higher than that for negative approaching behavior [TS alone condition:* p* = 0.011, *d* = 0.25, 95% CI = (5.2, 36.0); ICF condition: *p* = 0.002, *d* = 0.51, 95% CI = (24.9, 97.8)]. No significant differences were detected for the SICI condition.

**Table 1:** Motor evoked potential amplitudes (mV) for each TMS condition sorted by behavior

Emotion	Behavior	TS alone	SICI	ICF
Positive	Approaching	1.54 ± 0.86	1.16 ± 0.91	2.32 ± 1.36
	Avoiding	1.33 ± 0.80	0.96 ± 0.71	1.86 ± 1.17
Negative	Approaching	1.43 ± 0.78	1.07 ± 0.78	1.83 ± 1.16
	Avoiding	1.67 ± 1.03	1.24 ± 1.04	2.42 ± 1.37

The MEP ratios (% of baseline) sorted block are shown in Fig. 3b. The results of a two-way RM ANOVA showed significant main effects of TMS condition [*F*(1.424, 27.048) = 34.94, *p* < 0.001, ${\eta }_{p}^{2}$ = 0.65] and block [*F*(1, 19) = 16.44, *p* = 0.001, ${\eta }_{p}^{2}$ = 0.46]. The interaction of TMS condition and block was also significant [*F*(1.342, 25.499) = 5.67, *p* = 0.017, ${\eta }_{p}^{2}$ = 0.23]. Post-hoc analyses indicated that under the TS alone condition and the ICF condition, the MEP ratio of the automatic block was higher than that of the regulated block [TS alone condition: *p* = 0.006, *d* = 0.25, 95% CI = (6.6, 35.1); ICF condition: *p* = 0.002, *d* = 0.46, 95% CI = (6.6, 39.3)]. No significant difference was found for the SICI condition.

### Intracortical circuits

**Fig. 4:**
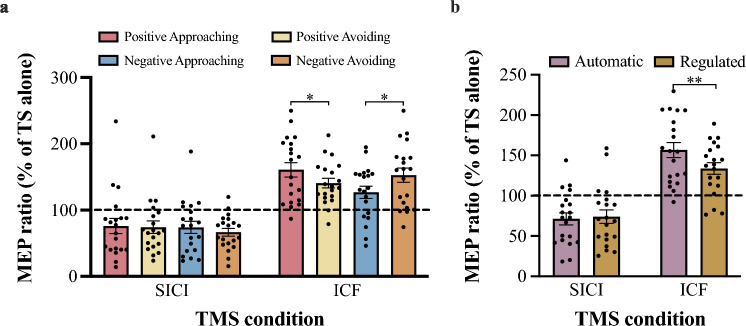
MEP ratio (% of TS alone) for each TMS condition sorted by behavior (**a**) and block (**b**). *Error bars* indicate SEM. *Dashed lines* indicate the MEP ratio for the TS alone condition (100%). Ratios <100% indicate inhibition, and ratios >100% indicate facilitation. ^*^p < 0.05; ^**^p < 0.01

For M1 intracortical circuits, the MEP ratios (% of TS alone) under pair-pulse conditions are shown in Fig. 4a. The results of a three-way ANOVA of MEP ratios revealed a significant main effect of TMS condition [*F*(1, 19) = 47.93, *p* < 0.001, ${\eta }_{p}^{2}$ = 0.72]. Paired-sample t-tests comparing the different TMS conditions indicated that the MEP ratios (% of TS alone) were significantly higher for the ICF (145.2 ± 32.8%) than for the SICI (72.7 ± 33.5%), confirming the effects of the paired-pulse protocol. No other significant main effects were observed [emotion: *F*(1, 19) = 1.60, *p* = 0.222, ${\eta }_{p}^{2}$ = 0.08; behavior: *F*(1, 19) = 0.04, *p* = 0.835, ${\eta }_{p}^{2}$ = 0.002]. There was a significant two-way interaction between emotion and behavior [*F*(1, 19) = 4.90, *p* = 0.039, ${\eta }_{p}^{2}$ = 0.21], and a significant three-way interaction [*F*(1, 19) = 7.85, *p* = 0.011, ${\eta }_{p}^{2}$ = 0.29]. Paired-sample t-tests indicated that the ICF effect of positive approaching behavior was significantly stronger than positive avoiding [*p* = 0.037,* d* = 0.49, 95% CI = (1.4, 39.2)], and the ICF effect of negative avoiding behavior was significantly stronger than negative approaching [*p* = 0.027,* d* = 0.58, 95% CI = (3.2, 48.1)]. No significant difference among the different behaviors was observed for the SICI effect.

The MEP ratios (% of TS alone) sorted by block are shown in Fig. 4b. The results of a two-way RM ANOVA based on block showed significant main effects of TMS condition [*F*(1, 19) = 47.93, *p* < 0.001, ${\eta }_{p}^{2}$ = 0.72] and block [*F*(1, 19) = 4.90, *p* = 0.039, ${\eta }_{p}^{2}$ = 0.21] and a significant interaction between TMS condition and block [*F*(1, 19) =  7.85, *p* = 0.011, ${\eta }_{p}^{2}$ = 0.29]. Post-hoc analyses indicated that under the ICF condition, the MEP ratio of the automatic block was higher than that for the regulated block [*p* = 0.008, *d* = 0.62, 95% CI = (−5.2, 20.9)]. No significant difference was observed under the SICI condition.

### Correlation analyses

**Fig. 5:**
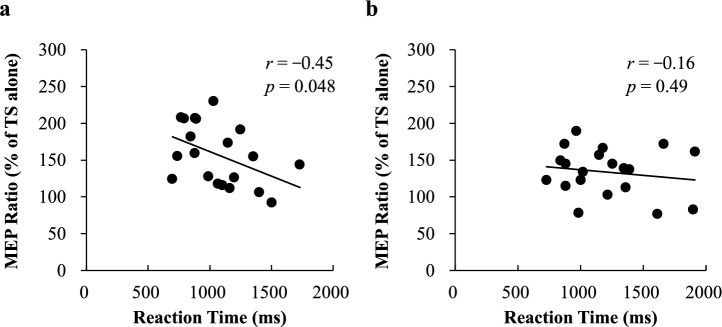
Correlation between RTs and MEP ratios (% of TS alone) during the intracortical facilitation condition for the automatic block (**a**) and regulated block (**b**)

Pearson correlation analysis showed a significant negative correlation between RT and ICF for the automatic block (*r* = −0.45, *p* = 0.048, Fig. 5a). There was no significant correlation for the regulated block (*r* = −0.16, *p* = 0.49, Fig. 5b).

## Discussion

This study used paired-pulse TMS protocols to explore inhibitory and facilitatory mechanisms in M1 during automatic action tendencies. We found (1) a stronger ICF effect during automatic (vs. regulated) behavior and (2) a significant negative correlation between RT and ICF during automatic behavior. However, no significant difference was detected for SICI between these two behaviors.

Consistent with the results of our previous studies, the present findings confirmed faster RTs for automatic behavior than for regulated behavior as well as greater corticospinal excitability when the TS was presented alone (Xia et al. 2021b, 2022). The dual-process model of social behavior explains social behavior as a joint function of reflective and impulsive processes (Strack and Deutsch 2004). The impulsive system elicits behavior based on associative links and motivational orientations through bottom-up processing, whereas the reflective system elicits behavior as a consequence of a decision process through top-down control from the prefrontal cortex (Ernst et al. 2013b, a; Bach et al. 2014; Bamford et al. 2015; Aupperle et al. 2015; Wallis et al. 2019). Here, these findings suggest that the representation of positive stimuli/negative stimuli is directly and tightly linked to the frequently co-occurring approaching/avoiding behaviors, resulting in a greater activation of the motor system to facilitate rapid automated behaviors. However, the MEP measured by single-pulse TMS (i.e., TS alone condition) could be considered as the net effect of both inhibitory and excitatory inputs to the descending corticospinal pathway, the underlying effect of cortical circuits is still unclear (Derosiere et al. 2020).

SICI and ICF are important markers of M1 cortical circuits. Previous studies assessing emotion perception have reported that fearful and happy signals modulate the motor system via selectively reducing the intracortical excitation in M1, as indexed by lower ICF compared with the neural signal, without affecting inhibitory cortical SICI (Borgomaneri et al. 2015b, 2017). In those studies, SICI and ICF were measured during the perception of the emotion, with no movement instruction. Thus, even when a fearful signal appeared, no overt behavior was allowed, which likely led to a lower facilitatory effect to inhibit the action tendency. We also observed the modulation of ICF rather than SICI in the present study, with a stronger ICF effect for automatic behavior compared with regulated behavior. This finding suggests that in regulated behavior, the weaker ICF effect could be related to inhibiting the action tendency while launching an alternative behavior. By contrast, behavior compatible with the action tendency (i.e., automatic behavior) leads to a greater facilitatory effect to speed the behavior. In addition, a significant correlation between RTs and the ICF was detected in automatic behavior, but not in regulated behavior, providing a potential mechanism for early motor facilitation during the processing of automatic behavior.

ICF is correlated with the magnetic resonance spectroscopy measurement of the glutamate concentration in M1 (Dyke et al. 2017), and glutamate is the main excitatory neurotransmitter in the central nervous system (Stan et al. 2014; Reddy-Thootkur et al. 2022). Based on the dual-process model, automatic behavior might mainly rely on fast bottom-up activities from subcortical structures (Ernst and Fudge 2009). Previous studies have found significant changes in glutamate concentrations in subcortical structures, such as the amygdala, during emotional processing, suggesting that functional activation of circuits related to emotional processing may be partially mediated by glutamatergic neurotransmission (Tran et al. 2013; Buchanan et al. 2016). The amygdala is reportedly in synergy with motor-related cortical areas, such as the supplementary motor area, with the signal subsequently transmitted to M1 through cortico-cortical pathways to regulate social behavior (Qin et al. 2012; Grèzes et al. 2013). In addition to functional connectivity, anatomical connections have also been found between the amygdala and M1, and the amygdala may also modulate more complex social behavior through a direct amygdala-motor pathway (Grèzes et al. 2014). Taken together, these studies have demonstrated that subcortical structures can modulate social behavior through distinct pathways to the motor cortex, which may modulate M1 glutamatergic circuits during automatic behavior. It has been reported that excessive release of glutamate is detected in individuals with drug use disorders, manifested by significantly higher increases in NMDA receptors in regions of the striatum, amygdala, and hippocampus after exposure to drug stimulation, suggesting that glutamate plays an important role in the activation of the automatic connection (Gass and Olive 2008). Hence, ICF may be useful for investigating the characteristics of abnormal motivational behavior and perhaps even as an effective indicator of such behaviors.

Previous studies have reported changes in SICI related to response inhibition, with greater GABAergic activity in M1 related to better inhibitory control suggesting that inhibitory circuits in the motor cortex are involved in stopping (Chowdhury et al. 2018, 2019a, b; He et al. 2019; Tran et al. 2020; Ding et al. 2021). However, different from those studies, we found no significant modulation of SICI in either automatic or regulated behaviors. Our finding may be because not only was response inhibition needed to suppress the automatic action tendency, which would be expected to increase SICI, but also the launch of an opposite behavior was required, which would decrease SICI (Hummel et al. 2009; Duque and Ivry 2009). It has been proposed that the strength of SICI is indicative of GABAergic efficacy within not only M1 but the frontal cortex more broadly, and that GABA_A_ receptors elsewhere in the frontal cortex are more directly responsible for the stopping response (Neubert et al. 2011; Chowdhury et al. 2019b). According to the dual-process model of social behavior, the execution of regulated behavior relies on the successful inhibition of automatic action tendencies, in terms of the neuroscientific explanation, the inhibition of subcortical bottom-up activity by prefrontal cortical top-down regulation (Ernst et al. 2013a, b). Hence, the null effect of SICI in the present study could be caused by GABAergic efficacy within both M1 and the prefrontal cortex.

## Conclusions

ICF was increased with no change in SICI during automatic behavior compared with regulated behavior, suggesting that an increase in ICF without a change in SICI may promote the rapid execution of automatic behaviors. Our findings suggest that excitatory glutamatergic interneuronal networks play an important role in mediating approaching-avoiding behavior, further supporting the intrinsic connection between emotional processing and the motor system. These results expand our understanding of the biological bases of automatic action tendencies and suggest that ICF may be used to explore the characteristics of abnormal motivational behaviors, such as the automatic approaching behavior toward drugs, alcohol, or high-calorie foods by individuals with drug or alcohol use disorders or eating disorders, respectively.

## Data Availability

The data that support the findings of this study are available from the corresponding author upon reasonable request.

## References

[CR1] Aupperle RL, Melrose AJ, Francisco A (2015). Neural substrates of approach-avoidance conflict decision-making. Hum Brain Mapp.

[CR2] Bach DR, Guitart-Masip M, Packard PA (2014). Human hippocampus arbitrates approach-avoidance conflict. Curr Biol.

[CR3] Bamford S, Broyd SJ, Benikos N (2015). The late positive potential: a neural marker of the regulation of emotion-based approach-avoidance actions?. Biol Psychol.

[CR4] Berkman ET, Lieberman MD (2010). Approaching the bad and avoiding the good: lateral prefrontal cortical asymmetry distinguishes between action and valence. J Cogn Neurosci.

[CR5] Bhattacharjee S, Kashyap R, Abualait T (2021). The role of primary motor cortex: more than movement execution. J Mot Behav.

[CR6] Borgomaneri S, Vitale F, Avenanti A (2015). Early changes in corticospinal excitability when seeing fearful body expressions. Sci Rep.

[CR7] Borgomaneri S, Vitale F, Gazzola V, Avenanti A (2015). Seeing fearful body language rapidly freezes the observer’s motor cortex. Cortex.

[CR8] Borgomaneri S, Vitale F, Avenanti A (2017). Behavioral inhibition system sensitivity enhances motor cortex suppression when watching fearful body expressions. Brain Struct Funct.

[CR9] Buchanan RJ, Gjini K, Modur P (2016). In vivo measurements of limbic glutamate and GABA concentrations in epileptic patients during affective and cognitive tasks: a microdialysis study. Hippocampus.

[CR10] Chen M, Bargh JA (1999). Consequences of automatic evaluation: immediate behavioral predispositions to approach or avoid the stimulus. Personal Soc Psychol Bull.

[CR11] Chowdhury NS, Livesey EJ, Blaszczynski A, Harris JA (2018). Variations in response control within at-risk gamblers and non-gambling controls explained by GABAergic inhibition in the motor cortex. Cortex.

[CR12] Chowdhury NS, Livesey EJ, Harris JA (2019). Individual differences in intracortical inhibition during behavioural inhibition. Neuropsychologia.

[CR13] Chowdhury NS, Livesey EJ, Harris JA (2019). Contralateral and ipsilateral relationships between intracortical inhibition and stopping efficiency. Neuroscience.

[CR14] Davranche K, Tandonnet C, Burle B (2007). The dual nature of time preparation: neural activation and suppression revealed by transcranial magnetic stimulation of the motor cortex. Eur J Neurosci.

[CR15] Derosiere G, Vassiliadis P, Duque J (2020). Advanced TMS approaches to probe corticospinal excitability during action preparation. Neuroimage.

[CR16] Ding Q, Cai H, Wu M (2021). Short intracortical facilitation associates with motor-inhibitory control. Behav Brain Res.

[CR17] Duque J, Ivry RB (2009). Role of Corticospinal Suppression during Motor Preparation. Cereb Cortex.

[CR18] Dyke K, Pépés SE, Chen C (2017). Comparing GABA-dependent physiological measures of inhibition with proton magnetic resonance spectroscopy measurement of GABA using ultra-high-field MRI. Neuroimage.

[CR19] Ernst M, Fudge JL (2009). A developmental neurobiological model of motivated behavior: Anatomy, connectivity and ontogeny of the triadic nodes. Neurosci Biobehav Rev.

[CR20] Ernst LH, Ehlis A-C, Dresler T (2013). N1 and N2 ERPs reflect the regulation of automatic approach tendencies to positive stimuli. Neurosci Res.

[CR21] Ernst LH, Plichta MM, Lutz E (2013). Prefrontal activation patterns of automatic and regulated approach–avoidance reactions—a functional near-infrared spectroscopy (fNIRS) study. Cortex.

[CR22] Faul F, Erdfelder E, Lang AG, Buchner A (2007). G*Power 3: a flexible statistical power analysis program for the social, behavioral, and biomedical sciences. Behav Res Methods.

[CR23] Fini C, Fischer M, Bardi L (2020). Support from a TMS/MEP study for a direct link between positive/negative stimuli and approach/avoidance tendencies. Neuropsychologia.

[CR24] Fischer M, Fini C, Brass M, Moors A (2020). Early approach and avoidance tendencies can be goal-directed: support from a transcranial magnetic stimulation study. Cogn Affect Behav Neurosci.

[CR25] Gass JT, Olive MF (2008). Glutamatergic substrates of drug addiction and alcoholism. Biochem Pharmacol.

[CR26] Grèzes J, Adenis M-S, Pouga L, Armony JL (2013). Self-relevance modulates brain responses to angry body expressions. Cortex.

[CR27] Grèzes J, Valabrègue R, Gholipour B, Chevallier C (2014). A direct amygdala-motor pathway for emotional displays to influence action: a diffusion tensor imaging study. Hum Brain Mapp.

[CR28] Hallett M (2000). Transcranial magnetic stimulation and the human brain. Nature.

[CR29] He JL, Fuelscher I, Coxon J (2019). Individual differences in intracortical inhibition predict motor-inhibitory performance. Exp Brain Res.

[CR30] Hummel FC, Steven B, Hoppe J (2009). Deficient intracortical inhibition (SICI) during movement preparation after chronic stroke. Neurology.

[CR31] Isaacson JS, Scanziani M (2011). How Inhibition shapes cortical activity. Neuron.

[CR32] Keel JC, Smith MJ, Wassermann EM (2001). A safety screening questionnaire for transcranial magnetic stimulation. Clin Neurophysiol.

[CR33] Krieglmeyer R, Deutsch R (2010). Comparing measures of approach–avoidance behaviour: the manikin task vs. two versions of the joystick task. Cogn Emot.

[CR34] Kujirai T, Caramia MD, Rothwell JC (1993). Corticocortical inhibition in human motor cortex. J Physiol.

[CR35] Lang PJ, Bradley MM, Cuthbert BN (1999). International affective picture system (IAPS): Instruction manual and affective ratings.

[CR36] Neubert F-X, Mars RB, Olivier E, Rushworth MFS (2011). Modulation of short intra-cortical inhibition during action reprogramming. Exp Brain Res.

[CR37] Qin S, Young CB, Supekar K (2012). Immature integration and segregation of emotion-related brain circuitry in young children. Proc Natl Acad Sci.

[CR38] Reddy-Thootkur M, Kraguljac NV, Lahti AC (2022). The role of glutamate and GABA in cognitive dysfunction in schizophrenia and mood disorders—a systematic review of magnetic resonance spectroscopy studies. Schizophr Res.

[CR39] Stan AD, Schirda CV, Bertocci MA (2014). Glutamate and GABA contributions to medial prefrontal cortical activity to emotion: Implications for mood disorders. Psychiatry Res Neuroimaging.

[CR40] Strack F, Deutsch R (2004). Reflective and impulsive determinants of social behavior. Personal Soc Psychol Rev.

[CR41] Tandonnet C, Garry MI, Summers JJ (2010). Cortical activation during temporal preparation assessed by transcranial magnetic stimulation. Biol Psychol.

[CR42] Tatti R, Haley MS, Swanson OK (2017). Neurophysiology and regulation of the balance between excitation and inhibition in neocortical circuits. Biol Psychiatry.

[CR43] Tran L, Lasher BK, Young KA, Keele NB (2013). Depletion of serotonin in the basolateral amygdala elevates glutamate receptors and facilitates fear-potentiated startle. Transl Psychiatry.

[CR44] Tran DMD, Chowdhury NS, McNair NA (2020). Linking cortical and behavioural inhibition: testing the parameter specificity of a transcranial magnetic stimulation protocol. Brain Stimul.

[CR45] Wallis CU, Cockcroft GJ, Cardinal RN (2019). Hippocampal interaction with area 25, but not area 32, regulates marmoset approach-avoidance behavior. Cereb Cortex.

[CR46] Xia X, Li Y, Wang Y (2021). Functional role of dorsolateral prefrontal cortex in the modulation of cognitive bias. Psychophysiology.

[CR47] Xia X, Wang D, Song Y (2021). Involvement of the primary motor cortex in the early processing stage of the affective stimulus-response compatibility effect in a manikin task. Neuroimage.

[CR48] Xia X, Wang D, Wang L (2022). Connectivity from ipsilateral and contralateral dorsolateral prefrontal cortex to the active primary motor cortex during approaching-avoiding behavior. Cortex.

[CR49] Xia X, Pi Y, Xia J (2023). Bilateral motor cortex functional differences in left-handed approaching–avoiding behavior. Psychophysiology.

[CR50] Ziemann U, Rothwell JC, Ridding MC (1996). Interaction between intracortical inhibition and facilitation in human motor cortex. J Physiol.

